# Dental caries in children and vitamin D deficiency: a narrative review

**DOI:** 10.1007/s00431-023-05331-3

**Published:** 2023-11-15

**Authors:** Teodoro Durá-Travé, Fidel Gallinas-Victoriano

**Affiliations:** 1 Department of Pediatrics, School of Medicine, University of Navarra, Avenue Irunlarrea, 1, 31008 Pamplona, Spain; 2 Navarrabiomed (Biomedical Research Center), Pamplona, Spain; 3 Department of Pediatrics, Navarra Hospital Universitary, Pamplona, Spain

**Keywords:** Antimicrobial proteins, Children, Dental caries, Enamel defects, Salivary flow Vitamin D deficiency

## Abstract

Dental caries represents one of the most prevalent health problems in childhood. Numerous studies have assessed that vitamin D deficiency is highly related to dental caries in primary and permanent teeth in children. The aim of this study is to elaborate a narrative review about proposed mechanisms by which vitamin D deficiency interacts with dental caries process in children. Vitamin D deficiency during pregnancy may cause intrauterine enamel defects, and through childhood is accompanied by insufficient activity of antibacterial peptides, decreased saliva secretion, and a low level of calcium in saliva.

*Conclusion*: In conclusion, vitamin D deficiency would increase the risk of caries in the primary and/or permanent dentition. Relationship between vitamin D deficiency and dental caries is evident enough for vitamin D deficiency to be considered as a risk factor for dental caries in children. Optimal levels of vitamin D throughout pregnancy and childhood may be considered an additional preventive measure for dental caries in the primary and permanent dentition.

## Introduction

Dental caries is a multifactorial disease and represents one of the most prevalent health problems in childhood. A recent meta-analysis study involving children from five continents indicates that the worldwide prevalence of dental caries in primary teeth (with a sample size of 80,405 individuals) was 46.2%, and the worldwide prevalence of dental caries in permanent teeth (with a sample size of 1,454,871 individuals) was 53.8% [1]. Nevertheless, childhood caries in developing countries was reported to be more frequent than in developed countries. This fact is possibly due to differences in personal (sociodemographic status, education, nutritional habits, oral hygiene, accessibility to dental services, etc.) and oral environmental factors (oral microbiota, salivary flow, inadequate fluoridation, frequent consumption of dietary sugars, etc.) [2–6].

Dental caries has been considered as an infectious disease—transmissible—caused by the action of oral microbial biofilms. Therefore, their colonization by cariogenic bacteria (*Streptococcus mutans*, *lactobacilli*, etc.) would constitute a substantial risk factor. The metabolism of dietary fermentable sugars, especially sucrose and lactose, by the oral microbiota gives rise to organic acids (lactic acid is the predominant end-product from sugar metabolism), and in the presence of a critical degree of acidity in the oral cavity (pH < 5.5) enamel is demineralized. Consequently, there is a greater porosity of the enamel, which allows the acids to diffuse more deeply evolving to the formation of cavities. Caries is characterized by progressive lesions that, if left untreated, will increase in size, affecting the dental pulp, and resulting in inflammation, pain and finally, necrosis and loss of teeth, and even spread of infection extra dentally [7, 8].

Saliva plays a protective role against caries since salivary flow, on one hand, facilitates the dilution and elimination of ingested fermentable sugars and, on the other hand, it has a neutralizing capacity (buffer effect) of the degree of acidity in the oral cavity. That is, once the sugars are removed from the mouth by swallowing and salivary dilution, the biofilm acids can be neutralized by the buffering action of saliva. In addition, if the salivary flow was sufficiently saturated with calcium and phosphorus ions, demineralization would be stopped and mineral redeposition (remineralization) would be favored [3].

Traditionally, the biological effects of vitamin D had been almost exclusively related to bone metabolism (calcium deficiency causes rickets in infants and osteomalacia in adults). Currently, it is known that most cells in the body, including odontoblasts (dentin-forming), ameloblasts (enamel-forming) and salivary glands, contain vitamin D receptors (VDR), and that the binding of vitamin D with its VDR (a nuclear transcription factor) modulates the expression of numerous coding genes (approximately 5–10% of the human genome) related not only to mineral metabolism but also to cell life cycle, immune response, and energy metabolism (genomic effects) [9–12].

The relationship between vitamin D deficiency and dental caries studies dates back to the 1920s. The study by Mellanby and Pattison, published in 1928 (*The action of vitamin D in preventing the spread and promoting the arrest of caries in children*), provided the first evidence that vitamin D deficiency is associated with dental caries in children and also verified that oral and/or dietary supplementation with vitamin D decreased the risk of caries [13]. Since then, several epidemiologic observational studies, systematic review and meta-analysis of controlled clinical trials have assessed how vitamin D deficiency is highly linked with dental caries in primary and permanent teeth in children (low vitamin D may facilitate demineralizing of teeth, in a similar way to its known action on bone, via reduced concentrations of calcium and phosphate ions), and have suggested that vitamin D exposure in early life may play a role in caries prevention [12, 14–26]. A recent clinical study has found a significantly lower incidence and severity of caries in children aged < 3 years with continued supplementation with vitamin D at least in the autumn–winter period after the 12th month of life [27]. Besides, there is evidence that infants born to vitamin D-deficient mothers have higher level of dental caries in their primary teeth compared with infants born from mothers without vitamin D deficiency [28–31], and that a higher maternal vitamin D intake during pregnancy would be associated to a lower risk of dental caries in the offspring [32]. In addition, a recent cohort study supports an inverse relationship between the risk of caries in permanent teeth in children (aged 6–10 years) and low prenatal vitamin D levels [33].

The aim of this study is to elaborate a comprehensive literature review (narrative review) about several different proposed mechanisms that would explain the role of vitamin D as a protective factor against dental caries in children. This review is based on an electronic search of literature performed by two independent researchers in the PubMed database of the US National Library of Medicine published between January 1985 and May 2023. The following specific keywords (Medical Subject Headings) were used alone or in combination for the search: vitamin D or vitamin D deficiency/insufficiency and antimicrobial proteins, children, dental caries, enamel hypoplasia, and salivary flow. Exceptionally, the study by Mellanby and Pattison, published in 1928, has been included due to its historical relevance [13].

## Intrauterine enamel defects

Primary teeth begin their formation as early as 6 weeks into embryonic life, and the development and maturity of the enamel and dentin begins during the second trimester and continues after child’s birth [34]. Vitamin D has a significant role in odontogenesis. The vitamin D-VDR complex modulates the transcription of genes encoding various structural proteins synthesized by odontoblasts (dentin syaloglycoproteins and dentinphosphoproteins) and ameloblasts (amelogenins and enamelin) that make up the unmineralized extracellular matrix. In addition, vitamin D induces the intrauterine mineralization of tooth dentin and enamel. Therefore, vitamin D has a significant role in forming oral hard tissue, comprising tooth enamel and dentin, and a deficiency in vitamin D may affect primary and permanent teeth development [11, 14, 15, 21, 35, 36].

There is suggestive evidence that vitamin D deficiency during pregnancy affects the formation and mineralization of primary teeth and results in developmental defects, such as dentin hypomineralization and enamel hypoplasia and/or hypomineralization in the offspring [29, 35, 37, 38]. In turn, because enamel defects involve the interruption or arrest of the dental matrix, deteriorated enamel has been related to caries risk in cohort studies [28, 39, 40].

In addition, a recent randomized clinical trial has shown that high-dose vitamin D supplementation during pregnancy (2400 IU/d from pregnancy week 24 to 1-week post-partum) was linked to lower risk of enamel defects in the offspring. This study showed that the prevalence of enamel defects in both deciduous and permanent dentition was lower in children whose mothers received high-dose vitamin D supplementation during pregnancy compared to the standard dose (400 IU/day). This suggests that prenatal supplementation with high doses of vitamin D appears to constitute a preventive intervention to reduce the prevalence of enamel defects and, consequently, the risk of childhood dental caries [41].

In other words, since vitamin D is involved in tooth development, vitamin D deficiency during pregnancy may cause defects in enamel and increase the risk of dental caries and, consequently, vitamin D control levels prior to conception may be important to reduce the risk of enamel defects in deciduous teeth and they should be controlled throughout pregnancy and after delivery.

## Antimicrobial proteins

Vitamin D acts as an immune system modulator both in the innate and adaptive immune systems. Vitamin D exerts its immunomodulatory activity on human adaptive immune cells: B and T lymphocytes (both resting and activated), monocytes and dendritic cells (maturation and production of cytokines, acid phosphatase and hydrogen peroxide, and prevent excessive expression of inflammatory cytokines), and polinuclear cell lines (neutrophil motility and phagocytic function) through its effects on the VDR [42, 43]. On the other hand, the immunological role of vitamin D in innate immunity is stimulating the arrangement of some antimicrobial proteins (AMPs) synthesized by barrier and immune cells involved in innate immune system, including the mucosal lining of the gastrointestinal system [44, 45]. In fact, the vitamin D-VDR complex modulates the transcription of genes of certain AMPs and, consequently, activates the synthesis of cathelicidin and, to a lesser extent, β2-defensin and hepcidina [42, 44, 45]. It means, cathelicidin is controlled by vitamin D which has both antimicrobial and antiendotoxin activity. It displays cationic and anphiphilic properties that allow them to depolarize and break the bacterial cellular membrane, and also displays chemotactic properties and plays a role in cellular autophagia. AMPs (cathelicidin is one of them) are host defense peptides of the innate immunity acting against several oral cariogenic microorganisms [14, 19, 21, 42, 46, 47].

Historically, a relationship between dental caries and *Streptococcus mutans* has been documented. In fact, many studies have shown that the development of caries in children is preceded by increased colonization with *Streptococcus mutans* [48]. In addition, some studies have found a relation between salivary antimicrobial protein levels and changes in streptococcal composition in dental plaque [46, 49, 50].

Clinical studies on saliva in school children have found, on the one hand, that the concentration of cathelicidin in saliva in patients with vitamin D deficiency was significantly lower compared to patients with normal vitamin D status [19] and, on the other hand, that caries-free children had significantly higher levels of cathelicidin in comparison with children with dental caries [49, 51, 52]. It means, the optimization of organic levels of vitamin D could contribute to improve the innate antibacterial defense and/or resistance against caries. In addition, some authors, have proposed its potential application (combination of AMPs) for the prevention and treatment of dental caries [47, 50].

## Salivary disfunction

Several studies have reported that there is a significant and positive correlation between serum and saliva 25(OH)D levels in young children [53] and adults [54]. In fact, it has been suggested that the assessment of 25(OH)D saliva levels to detect 25(OH)D levels in circulation could be a non-invasive alternative to serum testing. Obviously, this positive relationship means that if serum 25(OH)D level decreases, so will salivary level. Microradiography test have found that salivary glands are target cells for vitamin D; this means, they contain VDR and, therefore, would be susceptible to their genomic effects [55].

Experimental studies have shown that fluid and electrolyte secretion from salivary glands is directly dependent on vitamin D. In fact, vitamin D deficiency decreases salivary flow and alters its composition by reducing the amount of calcium ions [56, 57]. Although these studies have been conducted in animal models, they provide sufficient data about the effect of vitamin D on the quality and quantity of salivary flow, which could be extrapolated to the human body.

That is, on one hand, vitamin D deficiency causes a decrease in salivary flow, so diminishing its buffering effect against organic acids and, consequently, its protective role against caries. On the other hand, salivary flow is the natural vehicle of minerals necessary for continued tooth remineralization, and, therefore, a deficiency of vitamin D would lead to a significant reduction of minerals in saliva, this being ineffective to promote remineralization and, consequently, increasing the risk of caries in the primary and/or permanent dentition.

Currently, the US Endocrine Society recommendation in pregnant women is a daily oral cholecalciferol supplementation of 1500–2000 IU [58] which should begin as early as possible [59]. In addition, both the American Academy of Pediatrics [60], and the US Endocrine Society [58], and also the ESPGHAN (The European Society for Paediatric Gastroenterology Hepatology and Nutrition) [61] recommend that the adequate intake and recommended dietary allowances for children 0–1 and 1–18 year should be at least 400 and 600 UI/day, respectively. In other words, newborns should start taking a pharmacological oral cholecalciferol supplement of 400 IU daily as soon as possible, and it should be maintained during the first year of life to ensure an adequate supply of vitamin D. Children and adolescents who do not achieve the recommended dietary allowances of vitamin D, even with the intake of vitamin D-fortified foods, should take an oral cholecalciferol supplement of 600 IU daily. Obviously, patients at risk of vitamin D deficiency will have higher daily requirements [62].

## Conclusions

Dental caries is a complex multifactorial disease, and is not an example of a classic infectious disease; perhaps dental caries can be described best as a complex biofilm-mediated disease that can be mostly ascribed to behaviors involving frequent ingestion of fermentable carbohydrate and poor oral hygiene in combination with inadequate fluoride exposure.

**Fig. 1:**
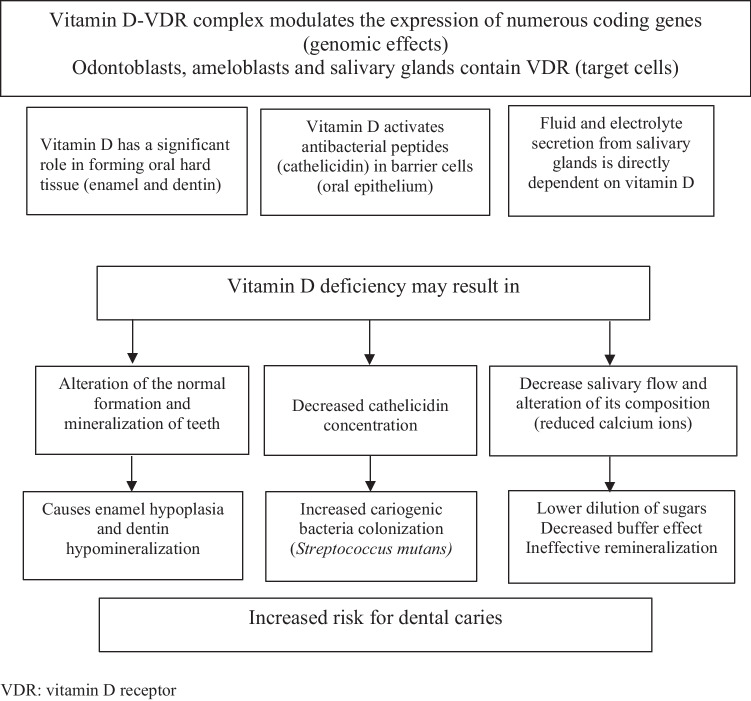
Proposed mechanisms by which vitamin D deficiency interacts with dental caries process. VDR, vitamin D receptor

Vitamin D deficiency during pregnancy may cause intrauterine enamel hypoplasia, and through childhood is accompanied by insufficient activity of antibacterial peptides, decreased saliva secretion, and a low level of calcium in saliva. In conclusion, vitamin D deficiency would increase the risk of caries in the primary and/or permanent dentition (Fig. 1).

Finally, it is important to indicate that the relationship between vitamin D deficiency and dental caries is evident enough for vitamin D deficiency to be considered as a risk factor for dental caries in children. Optimal levels of vitamin D throughout pregnancy and childhood may be considered an additional preventive measure for dental caries in the primary and permanent dentition. Oral health education campaigns to increase public awareness about the importance of vitamin D for oral health in children are highly recommended.

## Data Availability

The datasets generated during and/or analyzed during the current study are available from the corresponding author on reasonable request.

## Abbreviations

VDR: Vitamin D receptors
25(OH)D: 25-Hydroxicholecalciferol
AMPs: Antimicrobial proteins
